# Supplementary material for: Chlorovirus PBCV-1 Multidomain Protein A111/114R Has Three Glycosyltransferase Functions Involved in the Synthesis of Atypical N-Glycans
Source: Viruses. 2021 Jan 10;13(1):87. doi: 10.3390/v13010087 (PMC7826918; doi:10.3390/v13010087)
Supplement: Supplementary file 1 [file viruses-13-00087-s001.zip › Supplementary/Figure_S1.docx]

1 MTIVGITFGT EQYLGSAAVL RHSALTTGEF DEFRVFEKKD ISWLMDTYPN HFENSRGFGF

61 WCWKPFLIRN VMNQLPDGDV VVYCDSTMYF ERSIKPYIDH VEHNNPIVLC RLGGWSDNKN

121 DYRNRRWTKK SVFNSMGAGN TVAEEIQLNA SFQVYKNSPE TRAFVDQYLQ YCLNLDIIND

181 EGRDGGIFDT RHDQSILSIM ASEHPRVTFS RDVSQWGKQD PPCSISQPTG GAIELDALDE

241 NGVMHNLVNH HRRMMKIPKI AVITPTTGGK FLDACIKSVQ SSTLPNIEHW IIVDGREHEG

301 KVDMILEKYR HKHPVIKLVL PNNIGSGGWN GHRVFGSIPW IINADYISYL DDDNIVEPKH

361 FKDLVSSIIS TPNAKWSYCL RKLIDNNGNL IGYDNCESLG GISHTVAGRG DYLIDTSCYM

421 IDRDLAISAS PAWNARFRDP DGKPEPDREL SKCLLSAAPY GVVRKHSLNY RIGSTGLSVS

481 NNFFVQGNSI FGYDFEKYED IYVFHFSEKA TADFMNARRQ YKNRSYALEE WQMTLLKGLD

541 GMNGGKYNLL NGFTNFPNIP NKATILVSLC NPGDLPMDFF KERVDLQRIV YTLESPNIRH

601 QGQWNFNWLT QHFDVALTYF KPIIENKSIH TIFTPHNTHH GDLDDPHDAT ALLRVNKGVG

661 KSAGMILERR PHLFHTRDYA INGVHLRCLD YLREDLVRGL EDVTVFGINW GEVADGKKIK

721 LGHAKHRSQD ENSSVDLKSK FVFDIVVENC DAEGYVSEKL FDSLSAGCVP LYYGNMYDEL

781 GDLIPEGDVY FDLKKRNITT GKQLQELLDT LSDERVEGMR KNVIDYREKV LRFAGTKMFA

841 KKVEEAIELS KTTKKNVELV

**Figure S1.** Amino acid sequence of chlorovirus PBCV-1 encoded protein A111/114R. The A111/114R protein (NP_048459.2) has three putative GT domains: domain 1 (red, residues 1 to 260), domain 2 (black, residues 261 to 559), and domain 3 (blue, residues 560 to 860). Domain 1 is predicted to encode a GalT, domain 2 a XylT and domain 3 a FucT, based on homology modelling with other proteins. The signature GT motif DXD is underlined.
