# Supplementary material for: Chlorovirus PBCV-1 Multidomain Protein A111/114R Has Three Glycosyltransferase Functions Involved in the Synthesis of Atypical N-Glycans
Source: Viruses. 2021 Jan 10;13(1):87. doi: 10.3390/v13010087 (PMC7826918; doi:10.3390/v13010087)
Supplement: Supplementary file 1 [file viruses-13-00087-s001.zip › Supplementary/Table_S1.docx]

**Table S1.** Glycosyltransferase homologs of A111/114R domains obtained using Phyre2 analysis**.**

|  | **Ranking^1^** | **PDB code** | **Chain** | **Protein Name** | **Organism** | **Size^2^** | **Confidence^3^** | **% Coverage** | **% Identity** | **CAZy family^4^** |
| --- | --- | --- | --- | --- | --- | --- | --- | --- | --- | --- |
| **Domain 1** | 1 | 6BSV | B | Xyloglucan α-1,6-xylosyltransferase (XXT1) | *Arabidopsis thaliana* | 460 | 96.2 | 61 | 19 | GT34 |
|  | 2 | 5GVV | F | Galactosyltransferase (GlyE) | *Streptococcus pneumoniae* | 406 | 91.7 | 50 | 23 | GT8 |
|  | 3 | 6U4B | A | α-1,3-galactopyranosyltransferase / β-1,3-galactofuranosyltransferase (WbbM;RfbC) | *Klebsiella pneumoniae* | 630 | 91.6 | 50 | 14 | GT8/GT111 |
|  | 4 | 1LL2 | A | Glycogenin (Gyg) | *Oryctolagus cuniculus* | 333 | 90.7 | 77 | 20 | GT8 |
|  | 5 | 1GA8 | A | α-1,4-galactosyltransferase (LgtC) | *Neisseria meningitidis* | 311 | 90.3 | 59 | 14 | GT8 |
|  | 6 | 4WMA | A | Xyloside xylosyltransferase 1 (MGC) | *Mus musculus* | 392 | 90.2 | 82 | 12 | GT8 |
| **Domain 2** | 1 | 2Z86 | D | Chondroitin polymerase (K4CP) | *Escherichia coli* | 686 | 100 | 71 | 18 | GT2 |
|  | 2 | 5TZ8 | C | Teichoic acid β-N-acetylglucosaminyltransferase | *Staphylococcus aureus* | 573 | 100 | 97 | 15 | GT2 |
|  | 3 | 1XHB | A | α-N-acetylgalactosaminyltransferase (T1) | *Mus musculus* | 608 | 100 | 89 | 13 | GT27 |
|  | 4 | 6H4M | A | ss-1,3-N-acetylglucosaminyltransferase  (SA1808) | *Staphylococcus aureus* | 327 | 100 | 84 | 16 | GT2 |
|  | 5 | 6E4R | B | N-acetylgalactosaminyltransferase 9 | *Drosophila melanogaster* | 647 | 100 | 89 | 16 | GT27 |
|  | 6 | 6S24 | A | N-acetylgalactosaminyltransferase 3 | Taeniopygia guttata | 574 | 100 | 89 | 14 | GT27 |
| **Domain 3** | 1 | 2NZW | A | α-1,3-fucosyltransferase | *Helicobacter pylori* | 405 | 100 | 86 | 15 | GT10 |
|  | 2 | 4W6Q | C | Glucosyltransferase | *Streptococcus agalactiae* | 401 | 93.1 | 43 | 15 | GT8 |
|  | 3 | 3RHZ | B | Glucosyltransferase 3 | *Streptococcus parasanguinis* | 449 | 90.6 | 29 | 16 | GTnc^5^ |

^1^Models are ranked according to raw alignment score accounting sequence and secondary structure similarities

^2^Number of amino acids

^3^Confidence is the probability that the A111/114R-D1 sequence and the template are homologous

^4^CAZy Carbohydrate Active Enzyme website: www.cazy.org

^5^GTnc: glycosyltransferase family “not classified”
