# Supplementary material for: Chlorovirus PBCV-1 Multidomain Protein A111/114R Has Three Glycosyltransferase Functions Involved in the Synthesis of Atypical N-Glycans
Source: Viruses. 2021 Jan 10;13(1):87. doi: 10.3390/v13010087 (PMC7826918; doi:10.3390/v13010087)
Supplement: Supplementary file 1 [file viruses-13-00087-s001.zip › Supplementary/Figure_S2.docx]

D1 1 MTIVGIT---FGTEQYLGSAAVLRHSALTTGEFDEFRV--FEKKDISWLMDTYPNHFENSRGFGFWCWK-----------
GlyE 1 MRNTKRAVVFAGDYAYIRQ-------------------------------------------------------------
WbbM 1 MNSIKIYTCHHKPSAFLNASIIK---PLHVGKANTYNDIGCEGED-SGDNISFKNPFYCELTAHYWVWKNEPLADYVGFM
LgtC 1 MDIVF-----AADDNYAAY-------------------------------------------------------------


D1 65 --------------------------------------------------------------------------------
GlyE 20 --------------------------------------------------------------------------------
WbbM 77 HYRRHLNFAEQQNHPEDNWGVVNYPLINAEYESQFGLSDESISTCVDGYDLLLPKKWSVTSAGSKNNLDHYAKGEFLHIK
LgtC 15 --------------------------------------------------------------------------------


D1 65 --------------------------------------------------------------------------------
GlyE 20 -I------------------------------------------------------------------------------
WbbM 157 DYQSALDVVEELYPEYKDAIQQFNNATDGYYTNMFVMRKDMFTDYSEWLFAILSNLEDRISMNNYNAQEKRVIGHIAERL
LgtC 15 -L------------------------------------------------------------------------------


D1 65 --------------------------------------------------------------------------------
GlyE 21 -------------------------------------------------------ETAMKSLC-RH-N--SHLKIYLLNQ
WbbM 237 FNIYIIKSQQDKQLKIKELQRTFVTAETFNGKLNPIFDESVPVVISFDNNYALSGGALINSIV-RHSDANRNYDIVVLEN
LgtC 16 -------------------------------------------------------CVAAKSVEAAHPD--TEIRFHVLDA


D1 65 -------------------------------------PFLIRNVM----------NQLPDGDVVVYCDSTMYFERSIKPY
GlyE 42 DIPQEWFSQIRIYLQEMGG--DLIDCKLIGSQFQMNWSNKLPHINHMTFARYFIPDFVTE-DKVLYLDSDLIVTGDLTDL
WbbM 316 KVSHLNKQRLIK-LVAGHNNISLRFFD-VNSFTEMSDVHTRAHFSASTYARLFIPQLFREYKKVVFIDSDTVVKSDLATL
LgtC 39 GISEANRAAVAANLRGGGG--NIRFID-VNPEDFAGFPLNIRHISITTYARLKLGEYIADCDKVLYLDIDVLVRDSLTPL


D1 98 IDHVEHNNPIVLCRLGGWSDNK-------------------------------------NDYRNRRWTKKSVFNSMGAGN
GlyE 119 FELDLGENYLAAAR-SCFGAG-------------------------------VGFNAGVLLINNKKWGSETIRQK-----
WbbM 394 LDVEIGTNLVAAVK-DIVMEGFVKFGTMSESDDGIMPAGEYLKKTLGMTNPDEYFQAGIIVFNVEQMVKENTFAQ-----
LgtC 116 WDTDLGDNWLGACI-DLFVERQ-----------------EGYKQKIGMADGEYYFNAGVLLINLKKWRRHDIFKM-----


D1 141 TVAEEIQLNASFQVYKNSPETRAFVDQY---LQYCLNLDIINDEGRDGGI-------------------FD----TRHD-
GlyE 162 -----------------LID---LTEKEHENVEE-GDQSILNMLFKDQYSSLEDQYNFQIG-YDYGAATFK----HQFI-
WbbM 468 -----------------LMS---ALKAKK--YWF-LDQDIMNKVFFGRVKFLPLEWNVYHG-NGNTDDFFPNLKFSTYM-
LgtC 173 -----------------SCE---WVEQYKDVMQY-QDQDILNGLFKGGVCYANSRFNFMPTNYAFMANRFA----SRHTD


D1 194 -------QSILSIMASEH--PRVTFSRDV---------------------------------------------SQWGKQ
GlyE 215 ---FDIPLEPLP-LILHYISQDKPWNQFSVGRLREVWWEYSLMDWSVILNEWFSKSVKYPSKSQIFKLQCVNLTNSWCVE
WbbM 523 ---RFLEARRNP-KMIHYAGENKPWNTEKVDFYDDF-L-------ENV------------------------LNTPWEKE
LgtC 228 PLYRDRTNTVMPVAVSHYCGPAKPWHRDC---------------------------------------------TAWGAE


D1 220 DPP-------------------------------CSISQPT-GG------------------------------------
GlyE 291 KIDYLAEQLPEVHFHIVAYTNMANELLALTRFPNVT-VYPNSLPMLLEQIVIASDLYLDLNHDRKLEDAYEFVLKYKKPM
WbbM 567 IYY--------------------------RQLPVAT-VVPNQHTELQQTVLLQTKIK--------------------RAL
LgtC 263 RFT--------------------------ELAGSLT-TVPEEWRG-----------------------------------


D1 232 ------------AIELDALDENGVMHNLVNHHRRMM-------K---IPKI-
GlyE 370 IAFDNTCSENLSEISYEGIYPSSIPKKMVAAIRSYM-------R--------
WbbM 600 MPYVNKY------------APVGSPRRNKLTKY-YYKVRRSILG--------
LgtC 281 --------------------KLAVPHRMFSTKRMLQRW-RRKLSARFLRKIY

**Figure S2.** Amino acid sequence alignment of A111/114R-D1 and annotated GalTs. Invariant and similar residues are highlighted in black and gray, respectively. Sequences of GalTs from the following organisms were used (PDB code): *S. pneumoniae* GlyE (5GVV), *K. pneumoniae* WbbM (6U4B), and *Neisseria meningitidis* LgtC (1GA8). Multiple alignment was performed by T-Coffee [31] using structural information and homology extension. File output was compiled by BOXSHADE.
