# Supplementary material for: Chlorovirus PBCV-1 Multidomain Protein A111/114R Has Three Glycosyltransferase Functions Involved in the Synthesis of Atypical N-Glycans
Source: Viruses. 2021 Jan 10;13(1):87. doi: 10.3390/v13010087 (PMC7826918; doi:10.3390/v13010087)
Supplement: Supplementary file 1 [file viruses-13-00087-s001.zip › Supplementary/Figure_S3.docx]

**
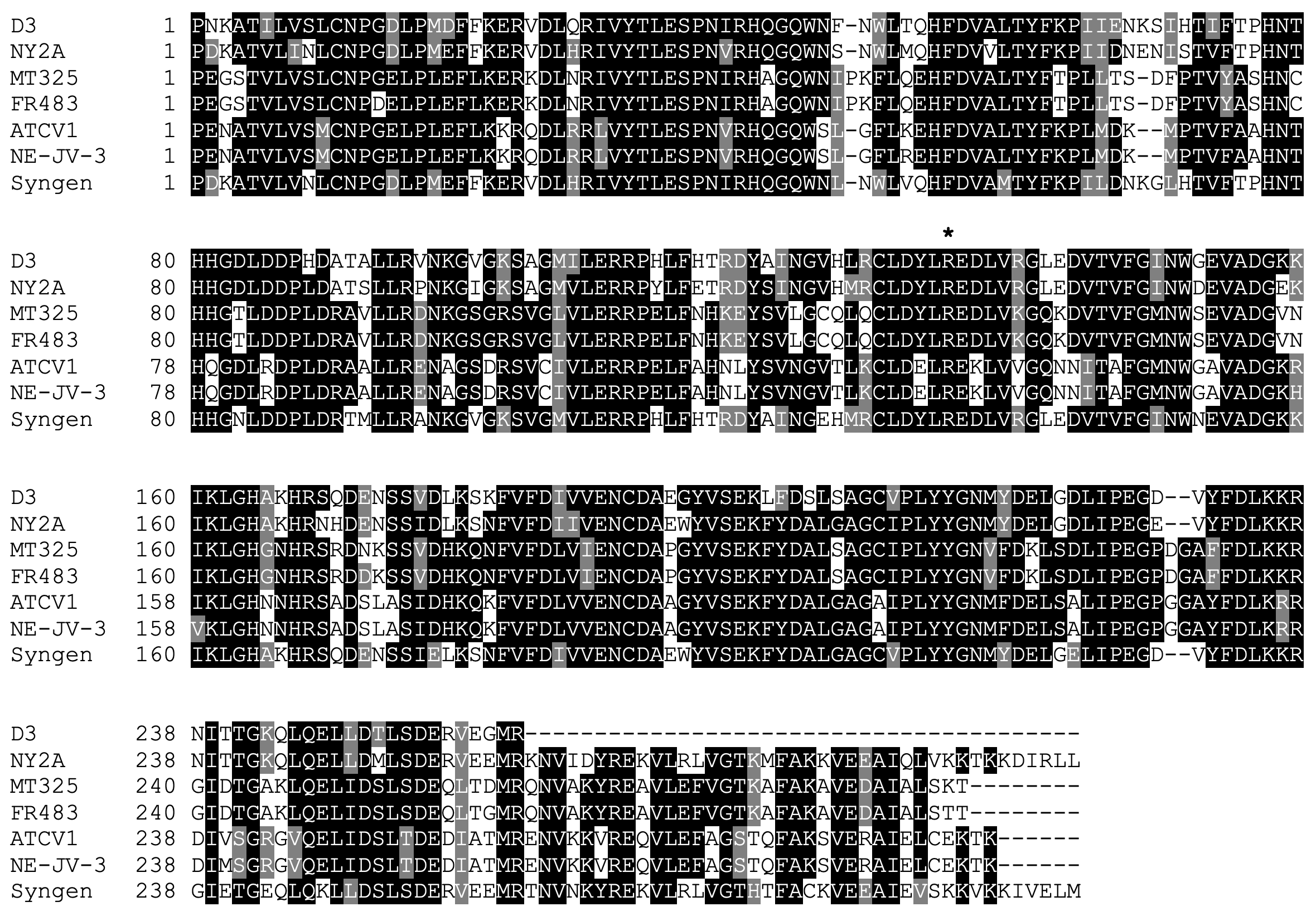
**

**Figure S3.** Amino acid sequence alignment of A111/114R-D3 and other chlorovirus orthologs. Invariant and similar residues are highlighted in black and gray, respectively. NC64A-chloroviruses PBCV-1 (A111/114R-D3) and NY2A (B159R); Pbi-chloroviruses MT325 (M467R) and FR483 (N472R); SAG-chloroviruses ATCV1 (Z120R) and NE-JV-3 (AGE56873.1:551-845); and Syngen-virus 5 (YP_009325562.1:560-860). The predicted residue essential for enzyme activity, Arg-693, is denoted with an asterisk. Multiple alignment was performed by T-Coffee [31] using structural information and homology extension. File output was compiled by BOXSHADE.
